# Maoberry (*Antidesma bunius*) ameliorates oxidative stress and inflammation in cardiac tissues of rats fed a high-fat diet

**DOI:** 10.1186/s12906-018-2400-9

**Published:** 2018-12-27

**Authors:** Arunwan Udomkasemsab, Chattraya Ngamlerst, Poom Adisakwattana, Amornrat Aroonnual, Rungsunn Tungtrongchitr, Pattaneeya Prangthip

**Affiliations:** 1 Department of Tropical Nutrition and Food Science, Faculty of Tropical Medicine, Mahidol University, 420/6 Ratchawithi Road, Ratchathewi, Bangkok Thailand; 2 Institute of Nutrition, Mahidol University, Nakhon Pathom, Thailand; 3 Department of Helminthology, Faculty of Tropical Medicine, Mahidol University, Bangkok, Thailand

**Keywords:** Cardiac tissue, Maoberry, Inflammation, Oxidative stress

## Abstract

**Backgound:**

Chronic fat-rich diets consumption is increased risk associated with cardiovascular diseases (CVD). Prevention or reduction the progression of cardiac tissue deterioration could benefit in CVD. This study aimed to examine the effects of maoberry (*Antidesma bunius*), a antioxidant-rich tropical fruit, supplementation on oxidative stress and inflammation in cardiac tissues of rats fed a high-fat diet (HFD).

**Methods:**

The male rats orally received HFD with maoberry extract doses of 0.38, 0.76 or 1.52 g/kg or simvastatin (10 mg/kg) for 12 weeks. At the end of the experimental period, the rats were fasted, euthanized and harvested for the hearts.

**Results:**

Significantly reduced oxidative stress (malondialdehyde levels) and enhanced antioxidant capacity (ferric-reducing activities) in cardiac tissues of the rats were found. Maoberry extract remarkably ameliorated the expressions of genes involved with pro-inflammatory such as the tumor necrosis factor alpha (TNF-α), interleukin-6 (IL-6), vascular cell adhesion molecule-1 (VCAM-1), monocyte chemoattractant protein-1 (MCP-1) and endothelial nitric oxide synthase (eNOS).

**Conclusions:**

Our findings suggest that maoberry extract has remarkable effects on preventing progression of cardiac tissue deterioration at least through lowering oxidative stress and inflammation.

## Background

Cardiovascular diseases (CVD) leading to heart failure are the main cause of mortality worldwide. Recent studies have indicated that a systemic inflammatory process can leads to the malfunctioning of the cardiac endothelium. Various mechanisms such as oxidative stress and inflammation are involved in cardiac pathogenesis. Therefore, the prevention or reduction of cardiac tissue deterioration to prevent CVD progression is considerable interested [1, 2].

Hypercholesterolemia is considered to be the hallmark of early CVD and is usually observed before vascular lesions appear. Previous studies have reported increased rates of reactive oxygen species (ROS) production in patients with hyperlipidaemia [3]. This may be attributed to fat-rich diets that increase the expression of nicotinamide adenine dinucleotide phosphate (NADPH) oxidase genes [4], which is the key enzyme responsible for ROS production [5], and that contributes to increased ROS formation in cells [6]. Overproduced ROS can react with nitric oxide (NO) to form peroxynitrite (ONOO^−^), a reactive short-lived peroxide, resulting in the inactivation of endothelial NO synthase (eNOS) [7], which lowers NO production and impairs vasodilatation, both of which can cause endothelial dysfunction [8–10]. Furthermore, oxidative stress contributes to CVD pathogenesis through inflammatory reactions. The lipid oxidation process originates from uncontrolled ROS overproduction, causing necrotic cell death, a major driver of inflammation [11]. In response to inflammation, monocytes and macrophages secrete pro-inflammatory cytokines such as the tumor necrosis factor alpha (TNF-α) and interleukin-6 (IL-6), stimulate the vasculatures to produce inflammatory mediators such as vascular cell adhesion molecule-1 (VCAM-1) and release chemokines such as monocyte chemoattractant protein-1 (MCP-1). All of these regulate the migration and infiltration of monocytes/macrophages into vascular inflammatory sites [2, 12]. The abundance of ROS causes low density lipoprotein (LDL) oxidation activating inflammatory cytokines, mediators and chemokines from infiltrating and resident macrophages. Both ROS and inflammation play a critical role in CVD occurrence and development [13].

Previous studies examined the beneficial effects of polyphenols, presenting in natural extracts of fruits, vegetables, soy, cocoa, tea and wine, and reported that these compounds can have biological effects on hearts [10, 14, 15]. The consumption of polyphenols as supplements appears to have various health benefits. Polyphenols present in red grape juice and red wine can markedly reduce NADPH oxidase activity and gene expression in human peripheral blood neutrophils and endothelial cells [15]. Patients with diabetes who daily consumed pomegranate as polyphenol supplements for 4 weeks showed a reduction in free radical-induced lipid peroxidation, notably through radical scavenger receptor activities [14]. Moreover, improvements in the regulation of pro-inflammatory molecules such as TNF-α, IL-6, VCAM-1 and MCP-1 [16–18].

Maoberry (*Antidesma bunius*) is a wild plant naturally growing throughout north-eastern Thailand. Maoberry fruits are very popular and are used in commercial products with healthy nourishment in Thailand. The fruits are considered to possess the ability to cure several ailments such as parched tongue, lack of appetite, indigestion, high blood pressure and diabetes [19, 20], possibly because of high polyphenol levels and antioxidant activity. *Antidesma* spp. contains several compounds such as phenolics, flavonoids, ascorbic acid and total proanthocyanidin [20–22]. *Antidesma* spp. also possesses strong antioxidant activity against oxidative damage with a variety of assays [21, 22].

Previously, we proposed maoberry extract benefit on atherogenic risk factors including lipid profiles, inflammation and oxidative stress in bloods [23]. To further understand the beneficial effects of maoberry extract on cardiovascular disease, we examined the effects of the maoberry extract on cardiac tissues in a hypercholesterol animal model. Oxidative stress and the specific molecules involved in endothelial damage were investigated. The results may provide additional data and encourage the application of natural extracts as an alternative for preventing damage in cardiac tissue.

## Methods

### Maoberry preparation

Dark-purple maoberry cultivars (*Antidesmabunius spp*.) were purchased from local orchard area of Khok Si Suphan District with Geocode of 4715, Sakon Nakhon province, North-eastern Thailand, during July and August in year 2016. The identification of maoberry cultivars were inspected confirmed by Assistant Dr. Prof. Pornprapha Chunthanom, Faculty of Natural Resources, Rajamangala University of Technology Isan, Sakon Nakhon campus, Sakon Nakhon, Thailand. After identifying, the fruits were washed, homogenized and concentrated via rotary evaporation method at 45 °C to a 40% concentration (*v*/v). The extracted juice was then aliquoted and preserved in opaque tubes at − 20 °C until used. A voucher specimen of maoberry has been deposited in Faculty of Natural Resources, Rajamangala University of Technology Isan, Sakon Nakhon campus.

### Animals and experimental settings

Five-week-old male Sprague Dawley rats, weighing 160–180 g, were obtained from the National Laboratory Animal Center at the Salaya Campus, Mahidol University. Seventy-eight rats were housed according to the rules and regulations of the Animal Care Ethical Committee of Laboratory Animal Science Center, Faculty of Tropical Medicine, Mahidol University (Approval no. FTM-ACUC 011/2018). All procedures were conducted according to the Guide for the Care and Use of Laboratory Animals published by the US National Institutes of Health. Two rats were housed per plastic cage in a controlled room (temperature, 25 °C ± 2 °C; relative humidity, 55% ± 10%; a 12-h light–dark cycle). After acclimatization for 1 week with free access to standard diet and drinking water, the rats were randomly separated into two groups with the same average weight. One group (*n* = 12; ND group) was fed a standard diet (3.90 kcal/g) containing 20.3% protein, 5% fat and 66% carbohydrate for 16 weeks, whereas the other group (*n* = 60; HFD group) were fed a high-fat diet (HFD; 5.40 kcal/g) containing 20.2% protein, 58.3% fat and 21.5% carbohydrate. Lard was the main fat component in HFD. After 4 weeks of feeding, the HFD rats were randomly divided into the following five subgroups (12 rats each) on the basis of the types of treatments they received (no differences in mean weight): HFD with distilled water (1.52 ml/kg, HF subgroup); HFD with maoberry extract (0.38 g/kg, ML subgroup, 0.76 g/kg, MM subgroup or 1.52 g/kg, MH subgroup) and 10 mg/kg simvastatin (STAT) by oral gavage every other day for 12 weeks.

### Tissue preparation

At the end of the experimental period, the rats were fasted for 12 h, following which they were euthanized by CO_2_ inhalation. The hearts were harvested and weighed. Cross-sections obtained from the middle of the heart were fixed on filter paper using 10% formalin buffer for at least 2 days. The remaining parts were snap frozen in liquid nitrogen and stored at − 80 °C. Each frozen heart part was exsanguinated by perfusion with cold normal saline (0.9%) and was placed in 100 mg/ml phosphate-buffered saline containing heparin. After cardiac tissues were completely lysed, they were homogenized on ice using a sonicator (Sonic Inc., Stratford, CT, USA) and were centrifuged at 10,000×g for 5 min at 4 °C. The supernatants were collected and stored at − 80 °C for further analysis.

### Determination of protein in cardiac tissues

The Bradford method (BioRad, Hercules, CA, USA) is rapid and accurate for estimating protein concentration. Following the manufacturer’s protocol with slight modifications, the dye reagent was prepared by diluting one part of dye reagent concentrate with four parts of distilled, de-ionized water. Bovine serum albumin (BSA) was used as the standard. The linear range of the assay for BSA is 0–2 mg/ml. Next, 160 μl of each standard and sample was pipetted into 96-well plate, and 40 μl of the diluted dye reagent was added to each well. The sample and reagent were thoroughly mixed using a microplate mixer. The final solution was incubated at room temperature for at least 5 min. Absorbance was measured at 450 and 595 nm. The eq. Y = 3.663X + 0.6762 (*r*^*2*^ = 0.9919) was used, and the results were recorded in g/L.

### Determination of total phenolic contents (TPC)

Total phenolic contents were determined using the Folin–Ciocalteu colorimetric method according to Baba and Malik (2015) [24] with slight modifications. In brief, 10 μl of cardiac tissue samples was blended with 150 μl of distilled water, mixed with 25 μl of Folin–Ciocalteu reagent and incubated for 3 min. Subsequently, 100 μl of 20% (*w*/*v*) sodium carbonate was added to each sample. The absorbance was measured at 650 nm after 1-h incubation in the dark at room temperature. A calibration curve was generated using gallic acid (10–100 μg/ml) solutions and the eq. Y = 0.0035X + 0.046 (*r*^*2*^ = 0.9999). The results were recorded as milligram gallic acid equivalences (mg GAE)/g protein. The solutions were assayed in duplicate.

### Determination of total flavonoid contents (TFC)

Total flavonoid contents were determined using the aluminium chloride colorimetric method [24] with some modifications. In brief, 1.5 μl of cardiac tissue sample was combined with 30 μl of methanol, thoroughly mixed with 120 μl of distilled water and treated with nine μl of 5% NaNO_2_ solution. After 5-min incubation, nine μl of 10% AlCl_3_ solution was added, and the mixtures were allowed to stand for 6 min. Next, 60 μl of 1 mol/L NaOH solution was added, and the final mixture was incubated for 15 min. Absorbance was measured at 410 nm. The calibration curve was generated using solutions of quercetin (100–1000 μg/ml) and the eq. Y = 8E–0.5X − 0.0025 (*r*^*2*^ = 0.9961). The results were presented in milligram quercetin equivalence (mg QE)/g protein. The solutions were assayed in duplicate.

### Oxidative stress in the heart

Thiobarbituric acid reactive substance (TBARS) assay was used for determining the level of malondialdehyde (MDA) by referring to lipid peroxidation from ROS. In brief, 100 μl of cardiac supernatant was thoroughly mixed with 350 μl of stock solution that contained 100 μl of 10% sodium dodecyl sulphate lysis solution and 150 μl of Thiobarbituric acid (TBA) reagent (25 ml of 10% acetic acid for 130 mg TBA) and was heated for 60 min at 95 °C. The absorbance of the supernatant was measured at 532 nm. MDA (#10009202, Cayman Chemical Company, Ann Arbor, MI, USA) was used as the standard. The standard curve (0–0.06 μmol/L of MDA) was derived using the following equation: Y = 0.0162X + 0.0097 (*r*^*2*^ = 0.9973). The results were presented as nmol/g protein.

### Ferric reducing antioxidant power (FRAP) assay

Ferric-reducing activities in the heart were determined using a modified FRAP assay [25]. In brief, the FRAP reagent was prepared in the dark using 300 mM sodium acetate buffer (pH 3.6), 10 mM 2,4,6-tri (2-pyridyl)-s-triazine solution in 40 mMHCl and 20 mM FeCl_3_ solution in a ratio of 10:1:1. The fresh working solution was warmed at 37 °C before use. Ten microliters of the cardiac tissue supernatant were allowed to react with 300 μl of the FRAP reagent. After incubation at 37 °C for 4 min, the absorbance of the reaction mixture was measured at 593 nm. A calibration curve was generated using the standard solutions of trolox (0–1000 μmol/L) and the eq. Y = 0.0011X − 0.0057 (*r*^*2*^ = 0.9917). The results were presented as μmol trolox equivalence (TE)/g protein.

### Histopathological analysis of cardiac tissues

Formalin-fixed cross-sections that were obtained from the middle of the heart were dehydrated and embedded in a paraffin–polyisobutylene mixture (Leica Biosystems, Harbourfront Centre, Singapore); 4-μm-thick sections were cut and prepared for haematoxylin and eosin (H&E) staining. The cardiac morphology was assessed using the Olympus BX-53 light microscope with a camera attachment (Tokyo, Japan) and the CellSens computer-based image analysis software (Olympus, Bangkok, Thailand).

### Real-time polymerase chain reaction

The molecular mechanism involved in the atherogenic protection of maoberry extract was determined by analyzing the mRNA levels of endothelial nitric oxide synthase (eNOS), tumor necrosis factor- alpha (TNF-α), interleukin-6 (IL-6), scavenger receptor CD36, vascular cell adhesion molecule-1 (VCAM-1) and monocyte chemoattractant protein-1 (MCP-1). Total RNA was isolated from frozen cardiac specimens using Trizol reagent (Invitrogen, Carlsbad, CA, USA, Cat. No. 15596–026) following the manufacturer’s instructions. The NanoDrop spectrophotometer (Thermo Fisher Scientific, Wilmington, DE, USA) was used to determine the concentration and purity of the extracted RNA. Total RNA (2 μg) was reverse transcribed into cDNA using random oligo (dT)18 primers (Thermo Fisher Scientific Inc., MA, USA) and reverse transcriptase enzyme (Thermo Fisher Scientific Inc., MA, USA). Polymerase chain reaction (PCR) using cDNA templates were performed in 10-μl reaction mixtures that contained 0.3 μl of each specific primer (10 μM), five μl of LightCycler® 480 SYBR Green I Master (Cat. No.04707516001) and 3.4 μl of RNase-free water. The reactions were run using the LightCycler® 480 Real-Time PCR detection system (Roche, Indianapolis, IN USA) with the following conditions: 95 °C for 5 min in the pre-incubation phase, 95 °C for 10 s, 58 °C for 10 s and 72 °C for 10 s and 45 cycles at the amplification phase. All primers (Table 1) were synthesized by Pacific Science Co., Ltd. (Bangkok, Thailand). The housekeeping gene glyceraldehyde-3-phosphate dehydrogenase (GAPDH) was used as the internal control to normalize for differences in quantity and quality between the RNA samples. The fold differences in the expression of different mRNAs were calculated and compared with those in that of the ND group using the 2^−ΔΔCt^ calculation as follows [26]:

$$ \Delta \Delta \mathrm{Ct}=\Delta \mathrm{Ct}\left(\mathrm{treatment}\ \mathrm{group}\right)-\Delta \mathrm{Ct}\left(\mathrm{normal}\ \mathrm{group}\right) $$

$$ \Delta \mathrm{Ct}=\mathrm{Ct}\left(\mathrm{target}\ \mathrm{gene}\right)-\mathrm{Ct}\left(\mathrm{reference}\ \mathrm{gene}\right) $$

**Table 1:** The primer details used for real-time PCR analysis

Genes	Forward sequence (5′ to 3′)	Reverse sequence (5′ to 3′)	Amplicon length (bp)	GenBank accession number
*eNos*	GGA TTC TGG CAA GAC CGA TTA C	GGT GAG GAC TTG TCC AAA CAC T	159	NM_021838.2
*Vcam-1*	GGA GCC TGT CAG TTT TGA GAA TG	TTG GGG AAA GAG TAG ATG TCC AC	105	NM_012889.1
*TNF-α*	ACT GAA CTT CGG GGT GAT TG	GCT TGG TGG TTT GCT ACG AC	153	NM_012675.3
*Il-6*	ATATGTTCTCAGGGAGATCTTGGAA	GTGCATCATCGCTGTTCATACA	80	NM_012589.2
*Cd36*	AGGAAGTGGCAAAGAATAGCAG	ACAGACAGTGAAGGCTCAAAGA	163	NM_031561.2
*Mcp-1*	GGC CTG TTG TTC ACA GTT GCT	TCT CAC TTG GTT CTG GTC CAG T	264	NM_031530.1
*Gapdh*	GCA AGT TCA ACG GCA CAG	GCC AGT AGA CTC CAC GAC AT	140	NM_017008.4

### Statistical analysis

The statistical analysis software SPSS (version 18.0; SPSS Inc., IBM, Chicago, IL, USA) was used to perform one-way analysis of variance and post-hoc tests for multiple comparisons. A *p* value of < 0.05 was considered to be significant. All values were expressed as mean ± standard deviation of two determinations. The GraphPad Prism software version 5.0 (La Jolla, CA, USA) was used for curve fitting.

## Results

### Total phenolic and flavonoid contents in cardiac tissues

**Fig. 1:**
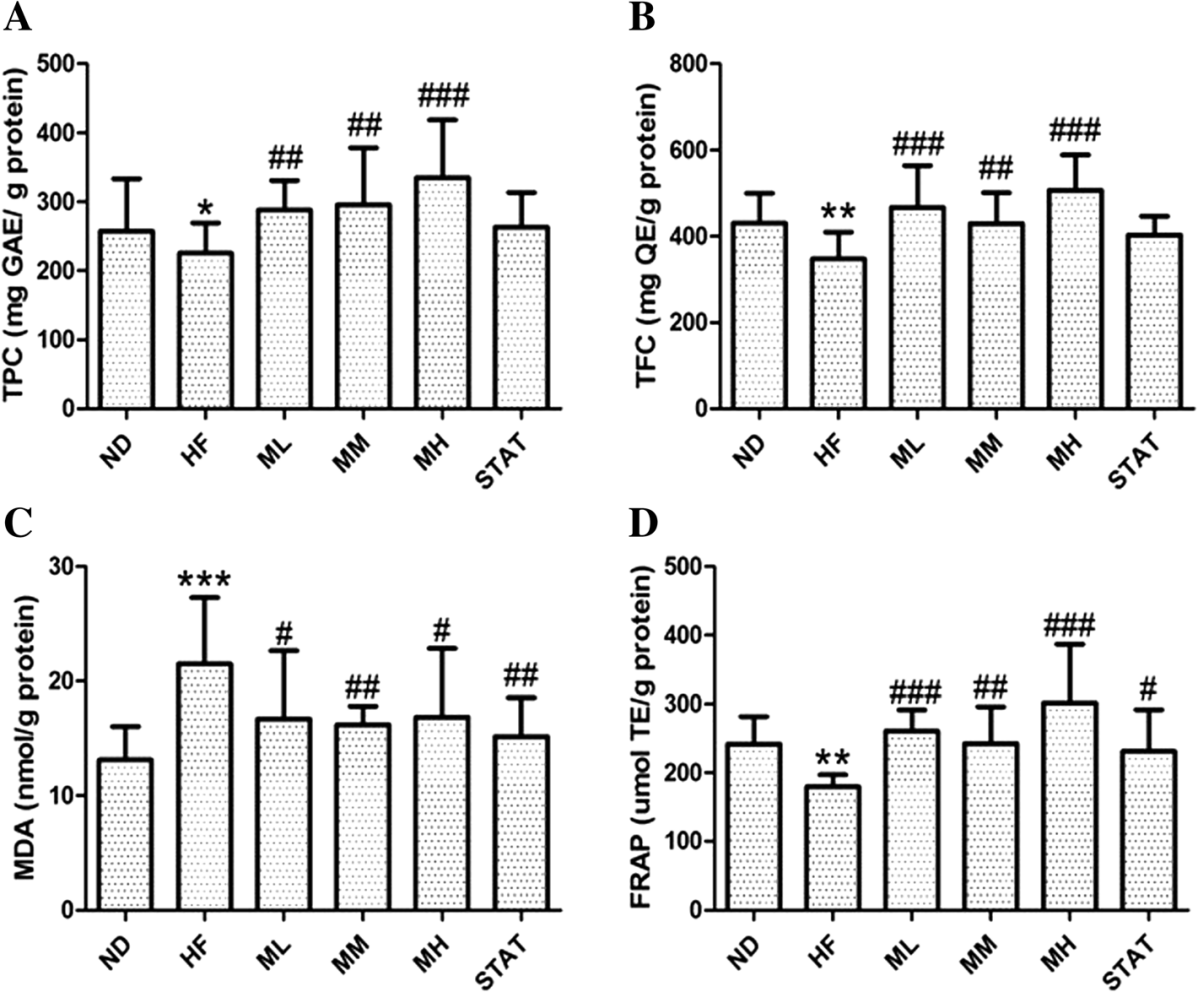
Total phenolic content (**a**), total flavonoid content (**b**), malondialdehyde level (**c**) and Ferric reducing antioxidant power (FRAP) level (**d**) in the cardiac tissue. Data are expressed as mean ± SD, *n* = 12. ND: standard diet, HF: high fat diet, ML, MM, MH: high fat diet with Mao Luangcrude extract 0.38 or 0.76 or 1.52 g/kg, respectively, STAT: high fat diet with simvastatin 10 mg/kg.*p < 0.05, **p < 0.01, ***p < 0.001 represent significant differences when compared with the ND group. #*p* < 0.05, ## *p* < 0.01, ### *p* < 0.001 represent significant differences when compared with the HF group

No mortality, illness and alterations in appearance or behavior were observed in all rats, both during and after 12 weeks of maoberry extract administration. A significant increase in both total phenols (*p <* 0.01 in the ML and MM subgroups and *p <* 0.001 in the MH subgroup) and total flavonoids (*p <* 0.01 in the MM subgroup and *p <* 0.001 in the ML and MH subgroups) were observed in cardiac tissues of rats that were fed HFD and different concentrations of maoberry extract compared with those of rats that were fed HFD with distilled water (Fig. 1a and b).

### Oxidative stress in cardiac tissues

Oxidative stress was evaluated on the basis of the production of MDA (a biomarker of lipid peroxidation) using the TBARS assay. After 12 weeks of treatment, MDA levels were significantly higher in the HF group than in the ND group (*p <* 0.001), confirming the establishment of oxidative stress in myocytes associated with hypercholesterolemia. Furthermore, MDA levels were significant lower in all rats that were fed HFD with maoberry extract (*p <* 0.05 in the ML and MH subgroups and *p <* 0.01 in the MM subgroup) or simvastatin (*p <* 0.01) than in rats that were fed HFD with distilled water (Fig. 1c).

### Antioxidant capacity in cardiac tissue

The FRAP level markedly decreased in the HF group compared with that in ND group (*p <* 0.01). In contrast, the FRAP levels significantly increased (*p <* 0.01 in the MM subgroup and p < 0.001 in the ML and MH subgroups) in rats that were fed HFD with maoberry extract or simvastatin (*p <* 0.05) compared with rats that were fed HFD with distilled water (Fig. 1d).

### Histopathological changes in cardiac tissue

**Fig. 2:**
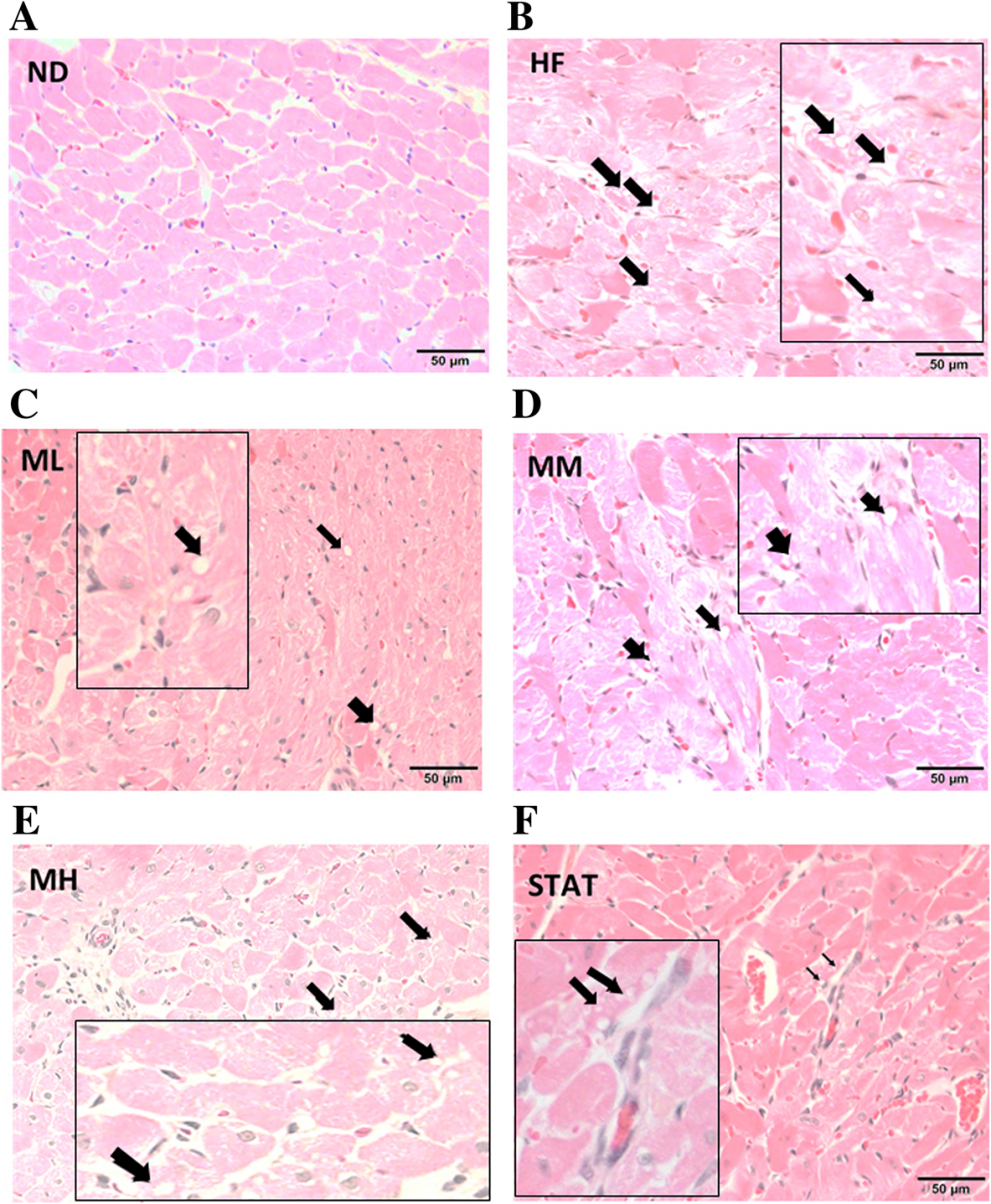
Light microscopic images of H&E-stained transverse-sections of cardiac tissues of rats after 12 weeks of Maoberry. For comparison, images showed different fat droplets in the different treatment groups (magnification × 100). **a** ND: rats fed a standard diet, **b** HF: rats fed a high-fat diet with distilled water, **c** ML, **d** MM and **e** MH: rats fed a high-fat diet with Maoberry extract at 0.38, 0.76 and 1.52 g/kg, respectively, **f** STAT: rats fed a high-fat diet with 10 mg/kg simvastatin. Arrows represent fat droplets. Panels enlarge the area of pathological alterations (magnifiacation × 400)

**Fig. 3:**
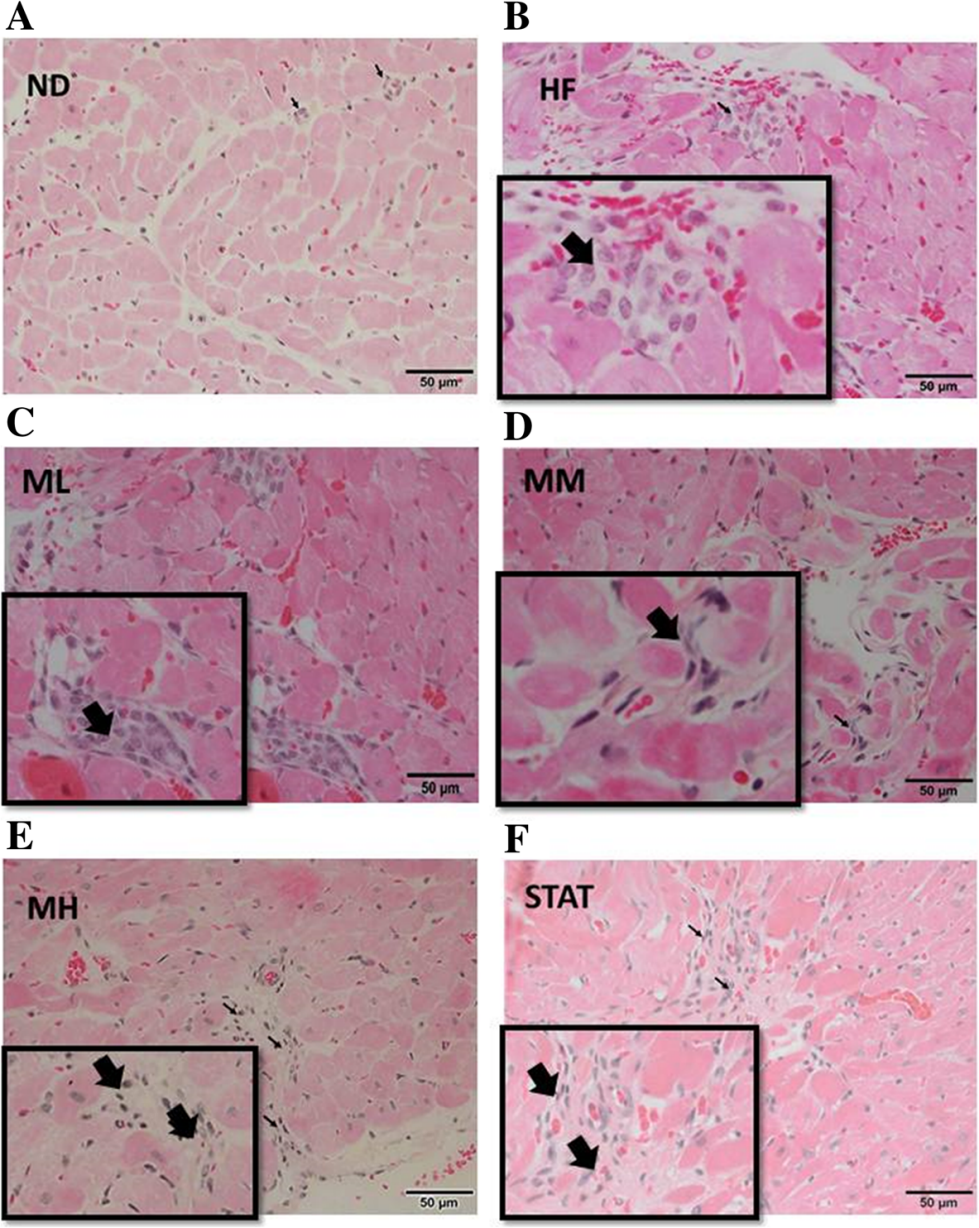
Pathological changes in transverse-sections of cardiac tissues of rats after 12 weeks of treatment showing amounts of mononuclear cell infiltration in different treatments. **a** ND: a standard diet, **b** HF: a high-fat diet with distilled water, **c** ML, **d** MM and **e** MH: a high-fat diet with Maoberry extract at 0.38, 0.76 and 1.52 g/kg, respectively, **f** STAT: a high-fat diet with 10 mg/kg simvastatin. H&E staining (magnification× 100). Arrows represent mononuclear cell infiltration. Panels enlarge the area of pathological alterations (magnifiacation × 400)

Microscopic images of H&E-stained cardiac tissues of rats in the ND group (Figs. 2a and 3a) revealed benign, blunt-looking, arranged cardiac muscles with no abnormal pathological findings. Alternatively, histological alterations were observed in cardiac tissues of rats in the HF group. Pathological alterations such as myocyte hypertrophy, enhancement of fat droplets and accumulation of mononuclear cells associated with inflammation were evident in the HF group compared with the ND group (Figs. 2b and 3b).

After 12 weeks of treatment, mild myocytic hypertrophy and a decrease in the number of fat droplets (Fig. 2c-e) and mononuclear cells (Fig. 3c-e) were evidently observed in Maoberry-treated rats, particularly those of the MH group, compared with those in HF group.

### Real-time PCR analysis of cardiac tissues

**Fig. 4:**
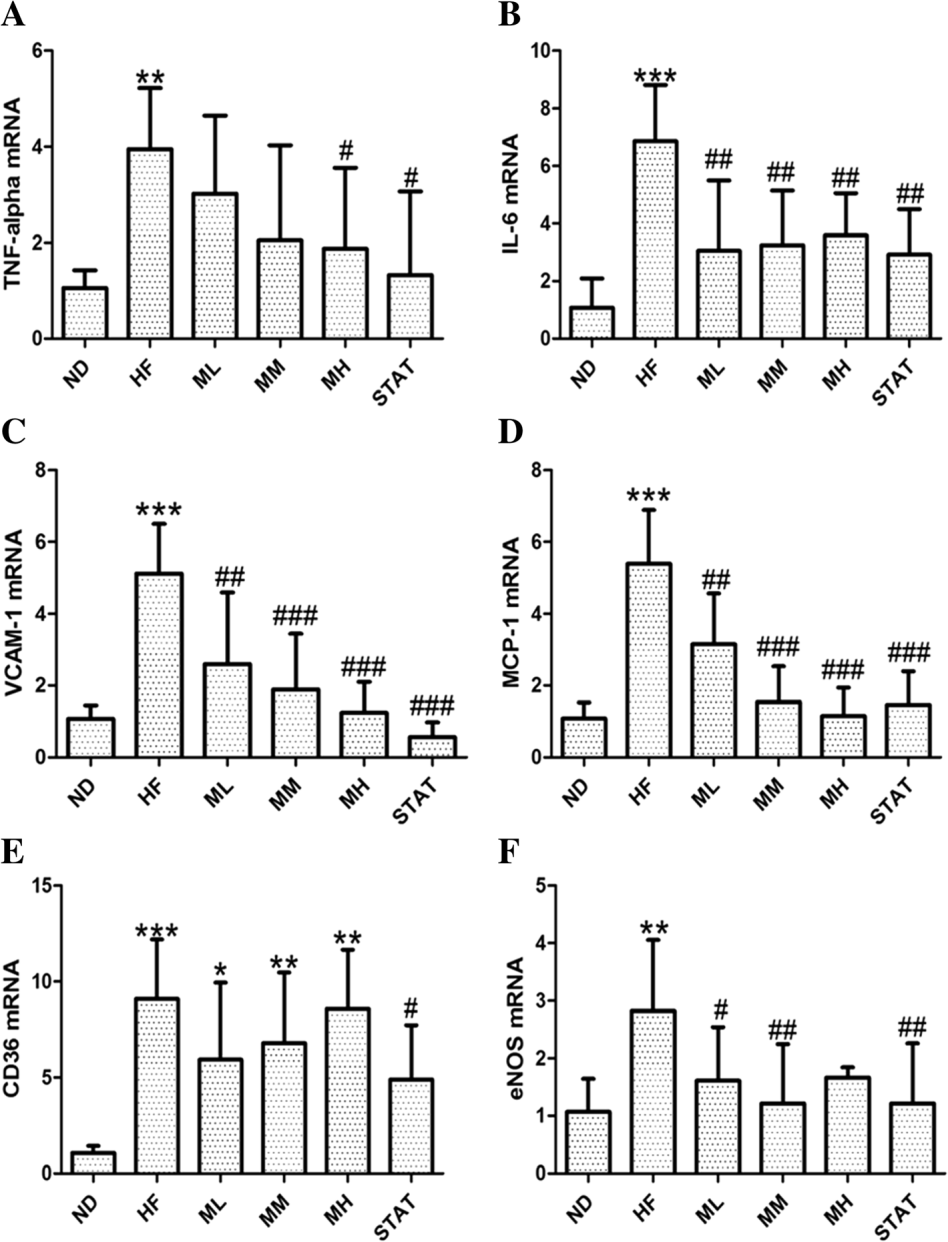
TNF-α, IL-6, VCAM-1, MCP-1, CD36 and eNOS mRNA levels from cardiac tissues of each groupby Real-time PCR detection. The expressions of TNF-α, IL-6, VCAM-1, MCP-1, CD36 and eNOS mRNA in HF group were significantly higher than those in the ND group. The expressions of IL-6, VCAM-1, MCP-1, and eNOS mRNA in Maoberrytreated groups were significantly lower than those in the HF group. Each bar represents the relative mean ± SD, n = 12 each. *p < 0.05, **p < 0.01 and ***p < 0.01 represent significant differences when compared with the ND group. #*p* < 0.05, ##*p* < 0.01 and ##*#p* < 0.001 represent significant differences when compared with the HF group. ND: a standard diet, HF: a high fat diet, ML, MM, MH: a high fat diet with Maoberry extract 0.38 or 0.76 or 1.52 g/kg, respectively, STAT: a high fat diet with simvastatin 10 mg/kg

To understand the effects of maoberry extract on HFD-induced inflammation and vascular dysfunction, mRNA levels were examined by real-time PCR analysis. The mRNA levels of inflammatory cytokines such as TNF-α and IL-6 were significantly elevated in the HF group (*p <* 0.01 and *p <* 0.001 respectively), whereas the levels were markedly decreased in rats that were fed HFD with different concentrations of maoberry extract or simvastatin (Fig. 4a and b).

Moreover, mRNA levels of VCAM-1, MCP-1, CD36 and eNOS were significantly higher in cardiac tissues of the HF group (*p <* 0.001, except eNOS, which was *p <* 0.01) than those of the ND group (Fig. 4c-f). Supplementing with different concentrations of maoberry extract or simvastatin significantly reduced the upregulation of VCAM-1, MCP-1 and eNOS mRNA levels compared with supplementing HFD with distilled water. However, significant downregulation in CD36 gene expressions was observed only in the STAT group.

## Discussion

According to our previous studies [23], maoberry extract doses of 0.38, 0.76 or 1.52 g/kg for 12 weeks may have tremendous beneficial effects on risk factors of cardiovascular disease such as atherogenic indices, inflammation and oxidative stress in peripheral blood and spleen histopathology.

The principle of maoberry dose use calculated according to American Heart Association recommendation to consume fruits 4 servings per day for long-term benefits to health and heart [27]. Four portions of fresh maoberries equal to 0.38 g per 1 kg body weight. Two and four times of maoberry extracts are 0.76 and 1.52 g/kg body weight, respectively. Previously, we examined nutritive values of maoberry extract and its antioxidant activities based on oxygen radical absorbance capacity (ORAC) and ferric reducing antioxidant power (FRAP) assays [23]. Gallic acid, epicatechin, catechin, and cyanidin-3-O-glucoside were analysed by HPLC and reported as the major polyphenolic components in fourteen maoberry cultivars from northeast Thailand [28]. Catechin, procyanidin B1, and procyanidin B2 analysed by HPLC are the major flavonoid compounds in fifteen cultivars from Northeast Thailand as well [29]. These polyphenols and flavonoids are often known to be antioxidants and may be involved in and contribute to the antioxidant activity of maoberry extract.

We previously reported that maoberry extract could reduce the number of immune cells associated with inflammation and the platelet population connected to the vascular blockage [23]. There are many finding on major active compounds from maoberry (*Antidesma bunius*) on oxidative stress and inflammation. Cyanidin-3-glucoside showed to inhibit free radicals and inhibit the expression of important cytokines in inflammation including nuclear factor-kappa B (NF-kB) in human vascular endothelial cells with dose-dependent manner [30]. Catechin showed to significantly reduce lipid peroxidation (MDA) in drug inducing cardiotoxicity in rats. Significant decrease in NF-kB, tumor necrosis factor alpha and inducible nitric oxide synthase were also present in rats treated with catechin [31]. Gallic acid available in maoberry was reported to be one of the most active dietary antioxidants in humans [32]. One of observation from Medical University of Vienna, Austria evidenced that administration of gallic acid in the amount of daily consumption could reduce oxidative DNA damage, oxidized-LDL and C-reactive protein in plasma of patients with type 2 diabetes mellitus [33].

The current study further demonstrates that maoberry extract contributes to prevent deterioration in cardiac tissue by improving the oxidative stress status and down regulating the expression of inflammatory cytokines and chemokines.

Oxidative stress, which occurs because of an imbalance between excess free radical formation and low antioxidant defense, has unspecific damage to the structure and functions of cells [34]. In the present study, a high fat diet (HFD) consumption appeared to increase lipid peroxidation in cardiac tissues, demonstrated by increased MDA levels in the tissues. MDA is a by-product of lipid peroxidation and is accepted as a vital marker of oxidative stress [35]. In previous studies in which animals were fed HFD, supplementation with lard induced oxidative stress, which originates from the upregulated expression of NADPH oxidase (up to threefold) [4, 8, 34]. After maoberry extract administration, numerous polyphenols in the extract pass through the stomach, are hydrolyzed and absorbed in the small intestine. Some polyphenols enter into the blood circulation and reach the organs [36]. In this study, although polyphenol levels in tissues depend on the amount of uptake and secretion by specific tissues, significantly higher levels of total flavonoids and total phenols were observed in the cardiac tissue of rats consuming maoberry extract. This meant that the cardiac tissue can uptake polyphenols from maoberry extract.

Furthermore, the results of the FRAP assay demonstrated the role of maoberry extract that reduces ferric iron (Fe^3+^) to ferrous iron (Fe^2+^) in the cardiac tissue. Altogether, these findings confirm that cardiac tissues can absorb polyphenolic substances, which are responsible for radical scavenging and reducing activities [37]. Similarly, polyphenols from other sources such as pomegranate [14], tea, cacao and red grape juice [15] are potential antioxidants that exert their effects by radical scavenging, which inhibits lipid peroxidation, Fe^3+^ reduction and downregulating NADPH oxidase activity [12, 15, 38].

Moreover, oxidative stress-induced LDL oxidation increases the expression levels of scavenger receptors and pro-inflammatory cytokines, mainly secreted by macrophages, and upregulates the expression of inflammatory adhesion molecules on endothelial cells [12, 34, 39]. In the current study, along with histopathological alterations, significantly elevated mRNA levels of TNF-α, IL-6, VCAM-1, CD36 and MCP-1 were observed in cardiac tissues of the HF group. Thus, it appears that the progression of endothelial dysfunction is associated with inflammation in the endothelium [40]. Inflammation reduced after 12 weeks of treatment with maoberry extract. A significant decrease in mRNA expression levels of pro-inflammatory cytokines and chemokines, such as TNF-α, IL-6 and MCP-1 and adhesion molecules, such as VCAM-1 was also observed. These results are buttressed by the apparent reduction in the number of lymphocytic cells in histological cardiac tissues of rats fed HFD with maoberry extract. In agreement with previous studies, the protective effects of polyphenols in maoberry extract may prevent endothelial dysfunction and inflammation via the downregulation of mRNA expression levels of TNF-α, IL-6, VCAM-1 and MCP-1 [18, 34, 39] by suppressing the activation of the transcription factor NF-κB in endothelial cells [41, 42]. In contrast, LDL oxidation enhances the expression of scavenger receptors such as CD36 in macrophages, which uptakes the excess modified lipid molecules, thus leading to the formation of foam cells, a key player in early inflammation-induced atherogenesis [43]. In the present study, we also found apparently high mRNA levels of CD36 in the HF group compared with those in the ND group, probably owing to the upregulation of structurally defined oxidized molecules, which serve as high affinity ligands for CD36 [43]. Although no significant difference was noted between the Mao Luang-treated groups and the HF group, mRNA expression levels of CD36 demonstrated a tendency to decline. Therefore, the beneficial effects of maoberry extract may be related to reduced CD36 expression and functions and suppressed oxLDL modification [39, 43]. Moreover, polyphenols in maoberry extract may suppress or downregulate the expression of other scavenger receptors that recognize oxidation-specific oxLDL, including SRA-1, SRA-2, MARCO, SR-B1, LOX-1 and PSOX [12].

Endothelial dysfunction is characterized by the impairment of endothelium-dependent relaxation owing to decreased vascular NO bioavailability caused by oxidative stress. eNOS is the major enzyme that is responsible for NO production. Lower eNOS levels or the lack of a substrate or cofactor such as L-arginine and tetrahydrobiopterin (BH4), which is affected by ROS, can lead to uncoupled eNOS formation, resulting in decreased NO levels and increased ONOO^−^ production. The overproduction of ONOO^−^ can enhance BH4 oxidation, leading to BH4 deficiency, thus generating a vicious circle. Furthermore, eNOS exposure to ONOO^−^ leads to the uncoupling of eNOS and is a crucial mechanism that contributes to vascular dysfunction [40, 44]. In the current study, we observed higher mRNA levels of eNOS in the HF group than in the ND group. After 12 weeks of treatment with maoberry extract, a reduction in upregulated mRNA levels of eNOS was observed, particularly in the ML (*p* < 0.05) and MM (*p <* 0.01) subgroups compared with HF group. This is in agreement with the results of a study on human umbilical vein endothelial cells, where in Genistein [45], a soy polyphenol, improved vascular reactivity by increasing eNOS expression. Genistein acutely stimulates eNOS synthesis in vascular endothelial cells. In contrast, Lund et al. [46] revealed that soy isoflavone did not affect eNOS expression in hyperlipidemic rabbits. These findings suggest that eNOS can be regulated by genomic and nongenomic factors [45]. Generally, regulatory systems allow living cells to change biochemical processes or gene expression programs automatically in response to alterations in the intracellular and/or extracellular environment. Therefore, in this study maoberry extract may affect on the modulation of eNOS expression into the normal range. Furthermore, during inflammation, TNF families alone could reduce eNOS mRNA half-life from 48 h to 3 h. [47]. Consequently, decreased mRNA levels of eNOS may indicate positive autoregulation related to inflammation, resulting in increased mRNA transcription and translation of eNOS [48], which were found in HF group. In contrast, maoberry extract may function as a free radical scavenger because of which eNOS and other substrates or cofactors can take effective action in NO production; negative feedback may control eNOS transcription and translation caused the decrease in eNOS mRNA expression [48]. Although the molecular mechanism remains unclear and complicated, the present study may be the first one to indicate that maoberry extract may prevent cardiac tissue deterioration.

## Conclusion

The current study reported for the first time that oral administration of maoberry extract had significant beneficial effects in cardiac tissues by reducing oxidative stress and enhancing antioxidant activity, thereby preventing progression of cardiac tissue deterioration involved in inflammation. In accordance with the outcomes of this study, maoberry extract ameliorates oxidative stress and inflammation in cardiac tissues of rats fed with a high-fat diet by at least through: (1) acting as a radical scavenger and Fe^3+^ reduction (2) reducing MDA levels that are associated with lipid peroxidation inside tissues and enhancing cardiac antioxidative capacity, (3) counteracting against the upregulation of inflammatory cytokines (TNF-α, IL-6, VCAM-1 and MCP-1) to reduce inflammation and (4) downregulating eNOS mRNA expression associated with positive and negative autoregulation.

Although the precise molecular mechanisms by which maoberry extract protects cardiac tissue deterioration remains unclear, this study may profoundly demonstrate that maoberry extract treatment may be a congenial alternative application against in damage progression of cardiac tissue.

## Abbreviations

BSA: Bovine serum albumin
eNOS: Endothelial NO synthase
FRAP: Ferric reducing antioxidant power
GAPDH: Glyceraldehyde-3-phosphate dehydrogenase
H&E: Haematoxylin and eosin
HFD: A high-fat diet
HFD: High-fat diet
IL-6: Interleukin-6
LDL: Low density lipoprotein
MCP-1: Monocyte chemoattractant protein-1
MDA: Malondialdehyde
MH: High-fat diet with maoberry extract 1.52 g/kg
ML: High-fat diet with maoberry extract 0.38 g/kg
MM: High-fat diet with maoberry extract 0.76 g/kg
NADPH: Nicotinamide adenine dinucleotide phosphate
ND: Normal diet
NO: Nitric oxide
ONOO^−^: Peroxynitrite
ROS: Reactive oxygen species
STAT: High-fat diet with simvastatin
TBA: Thiobarbituric acid
TE: Trolox equivalence
TFC: Total flavonoid contents;TBARS, Thiobarbituric acid reactive substance
TNF-α: Tumor necrosis factor alpha
VCAM-1: Vascular cell adhesion molecule-1
